# Sensitization to food allergens is associated with more severe wheezing in children 

**DOI:** 10.5414/ALX02546E

**Published:** 2024-12-31

**Authors:** Ozge Yılmaz, Cecilia M. Patino, Fatma Taneli, Esra Toprak Kanik, Ahmet Turkeli, Ceyhun Gozukara, Sezen Irmak, Hasan Yuksel

**Affiliations:** 1 Manisa Celal Bayar University, School of Medicine, Department of Pediatric Allergy and Pulmonology, Manisa, Turkey,; 2 University of Southern California, Keck School of Medicine, Department of Preventive Medicine, Los Angeles, CA, USA, and; 3 Manisa Celal Bayar University, School of Medicine, Department of Biochemistry, Manisa, Turkey

**Keywords:** food allergy, wheezing, surfactant protein D, club cell 16

## Abstract

Aims: We investigated sensitization to food allergens as a prognostic factor for wheezing in children with recurrent wheezing and compared serum club cell 16 (CC16) and surfactant protein D (SP-D) among these children with and without sensitization to food allergens. Materials and methods: Children with recurrent wheezing were enrolled in this prospective cohort study. Specific IgE to five common food allergens (Fx5) was assessed at baseline, and children were followed-up for 1 year for new-onset wheezing episodes. Baseline wheezing severity score, CC16, and SP-D levels were measured. Results: We enrolled 295 children (44 Fx5(+)). Poisson regression analysis revealed that Fx5 positivity changed the yearly frequency of wheeze by a factor of 1.66 (p = 0.05, 95% CI: (0.99 – 2.75)). Levels of CC16 and SP-D were not significantly different between the groups (p = 0.679 and p = 0.988). Conclusion: Sensitization to food allergens irrespective of food associated clinical allergy findings is associated with worse prognosis of wheezing in children.

## Introduction 

Wheezing is a common problem in children especially under 3 years of age [1, 2]. Environmental factors such as respiratory viral infections and allergen exposure as well as genetic predisposition are associated with persistence and clinical severity of wheezing as well as airway hyperreactivity [2]. Epithelium signal communication with the underlying structural cells has been defined as the epithelial mesenchymal trophic unit (EMTU), and this has been proposed to promote a microenvironment that facilitates allergic sensitization resulting in different types of inflammation in different organs. Epithelial barrier defects in different organs may underlie the coexistence of different allergic diseases [3]. Epithelium has two different functions: it is a barrier built by tight junctions between the cells, and it is also an element of the innate immune system. The genetic and environmentally induced defects in the epithelial barrier are associated with development of allergies and airway hyperreactivity [4]. Club cell 16 (CC16) is an immunosuppressor and anti-inflammatory molecule that inhibits neutrophil chemotaxis and phagocytosis, and it has been proposed to be a marker of epithelial barrier permeability [4]. Surfactant protein D (SP-D) is a lung collectin that increases in response to inflammation [5]. 

Food allergies have been reported to be associated with asthma, and the frequency of food allergy in asthmatic individuals was reported to be 2 – 12% [6, 7]. There are many potential mechanisms to explain this association, such as the immune deviation towards the Th2 phenotype in these children. Moreover, cross-reactivity of food allergens with inhalant allergens may lead to respiratory symptoms in children with food allergy [6]. 

We hypothesized that prognosis of wheezing in children with sensitization to food allergens may be different from those without food hypersensitivity. Therefore, in this study we aimed to investigate the influence of sensitization to food allergens irrespective of food-associated clinical allergy findings on the prognosis of wheezing during follow-up in young children diagnosed with recurrent wheezing and to evaluate if sensitization to food allergens is associated to more severe wheezing episodes in these children. Moreover, we hypothesized that the epithelial barrier defect associated with gastrointestinal sensitization to food allergens may also be associated with airway hyperreactivity, wheezing, and asthma. Thus, we aimed to evaluate if serum levels of CC16 and SP-D, as markers of lung epithelial permeability and lung inflammation, respectively, are different among wheezing children with and without sensitization to food allergens. 

## Materials and methods 

### Study design and ethics 

This is a prospective cohort study. Specific IgE to five common foods allergens was assessed at baseline, and the subjects were followed-up for 1 year for new-onset wheezing episodes. 

This study was approved by Celal Bayar University Institutional Review Board (2011-186), and written informed consent was obtained from the parents of all enrolled subjects. 

### Study population 

**Figure 1:**
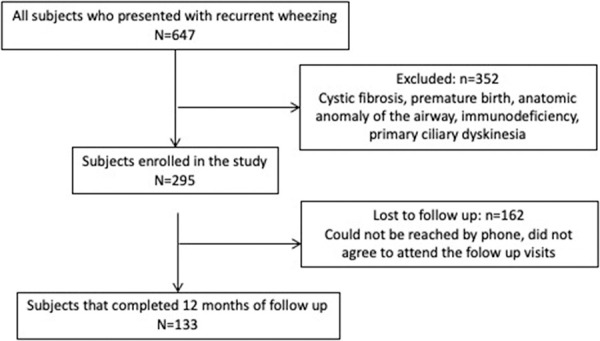
Flow diagram of the study (patient source and loss to follow-up).

All subjects with recurrent wheezing who presented to the Pediatric Allergy and Pulmonology outpatient department between September 2011 and December 2012 were enrolled consecutively in the order of presentation to the department (Figure 1). 

Inclusion criteria: 1) Children between 1 and3 years of age; 2) recurrent wheezing: 3 or more episodes of wheezing documented by a physician during the child’s life-time. 

Exclusion criteria: 1) Any other lung disease such as cystic fibrosis (CF), bronchiolitis obliterans, primary ciliary dyskinesia; 2) congenital cardiac disease; 3) immunodeficiency. 

### Study variables 

**Demographics**

Exposure: Food specific IgE (levels ≥ 0.35 kU/L were classified as sensitized). 

Primary outcome: Number of parent-reported wheezing episodes assessed every 3 months for 1 year. Wheezing was described to the parents by the study physician. 

Secondary outcomes: Number of parent-reported and wheezing-related number of emergency visits, hospitalizations, systemic steroid use (days), bronchodilator use (days). All secondary outcomes were assessed every 3 months for 1 year. SP-D levels and CC16 protein levels were assessed at baseline. 

Confounders: Family history of allergy and wheezing, total serum Ige, skin prick test for inhaled antigens. 

### Study procedures 

Measurement of serum food allergen level: Serum for measurement of Fx5 levels was obtained from all enrolled children. Allergen-specific IgE antibodies were measured using a mix of common food allergens (Fx5: hen’s egg, cow’s milk, peanut, soybean, wheat flour, and codfish). Standardized methods with the CAP System FEIA (Pharmacia Diagnostics, Freiburg, Germany) were used. Sensitization was defined as an IgE antibody level of 0.35 kU/L [8]. 

Measurement of serum SP-D: Serum samples for measurement of SP-D levels were obtained at enrollment from 194 children. They were measured with the enzyme-linked immunosorbent assay (ELISA) according to the kit manufacturer’s instructions (Biovendor RD194059100, Brno, Czech Republic). 

Measurement of serum CC16 levels: Similarly, serum samples for measurement of CC16 levels were obtained at enrollment from 194 children. Serum CC16 concentrations were measured using a commercially available ELISA method (Biovendor RD191022200). 

Recording of wheezing parameters: Wheezing severity at enrollment was evaluated by the researcher considering respiratory rate, retractions, and extent of wheezing [9]. 

All participants were informed about the follow-up period, and they were instructed to present to the outpatient department of the Pediatric Allergy and Pulmonology Department when the child experienced wheezing. Their presenting symptoms and lung auscultation findings were recorded by the researcher upon presentation. Participants were also asked to present to the outpatient department at the end of the 3-month period after the last visit even if no wheezing was detected. The patients who did not show up for follow-up were contacted by telephone and questioned about any wheezing episodes. Other prognostic variables including number of wheezing episodes, number of emergency visits for wheezing, number of days of systemic steroid use and number of days of bronchodilator use every 3 months were recorded for 1 year by the physician. 

### Sample size calculation 

A Poisson regression of the dependent variable, number of wheezing episodes in 1 year, on the binary independent variable, sensitization to food allergens, with proportion = 0.15 using a sample of 300 observations achieves 73% power at a 0.05 significance level to detect a response rate ratio of at least 1.5 due to a 1-unit change in the independent variable. The baseline rate is 1.0 and the mean exposure time is 1 year. The sample size was adjusted, since a multiple regression of the covariate of interest on the other covariates in the Poisson regression is expected to have an R^2^ of 0.03. 

### Data analysis 

To describe the study population age, gender, number of siblings, baseline wheezing, levels of serum SP-D levels and CC16 protein levels were compared across Fx5(+) and Fx5(–) groups using the independent sample’s t test and χ^2^. 

Poisson regression was used to model the association between status of sensitization to food allergens (yes vs. no) and primary and secondary clinical outcomes (number of wheezing episodes in the last year and clinical severity of wheezing episodes in the last year, number of emergency visits, number of hospitalizations, number of systemic steroid use, number of bronchodilator use). Relative risks, confidence intervals, and p-values were estimated. To evaluate that the model fit we assessed the assumptions of the Poisson model. 

## Results 

### Sociodemographic characteristics of the study population 

We enrolled 295 children (202 male) into this study; loss to follow-up was 55%, and 133 children remained in the cohort at the end of 12 months follow-up (Figure 1). 

At enrollment, the mean age of the Fx5(+) group was 20.4 ±9.5 months and that of the Fx5(–) group was 17.8 ± 8.9 months (p = 0.09). Sex was not significantly different between the Fx5(+) and Fx5(–) groups (p = 0.86). Mean numbers of siblings were 0.9 ± 0.8 vs. 0.7 ± 0.8 in the Fx5(+) and Fx5(–) groups, respectively (p = 0.05) (Table 1). 

Family history of allergy was present in 38.6% of the Fx5(+) subjects and in 47.6% of the Fx5(–) subjects (p = 0.33). 

### Wheezing severity parameters in the study groups 

There was no significant difference between the groups when wheezing severity at baseline and enrollment into the study was considered (3.1 ± 2.8 vs. 2.9 ± 2.5, p = 0.58). Similarly, indicators of wheezing prognosis during the 1-year follow-up period such as the total number of wheezing episodes, number of physician and emergency visits, or the total number of days of bronchodilators use were not significantly different between children with and without Fx5 positivity (p = 0.69, p = 0.43, p = 0.46, p = 0.99, respectively) (Table 1). 

**Figure 2:**
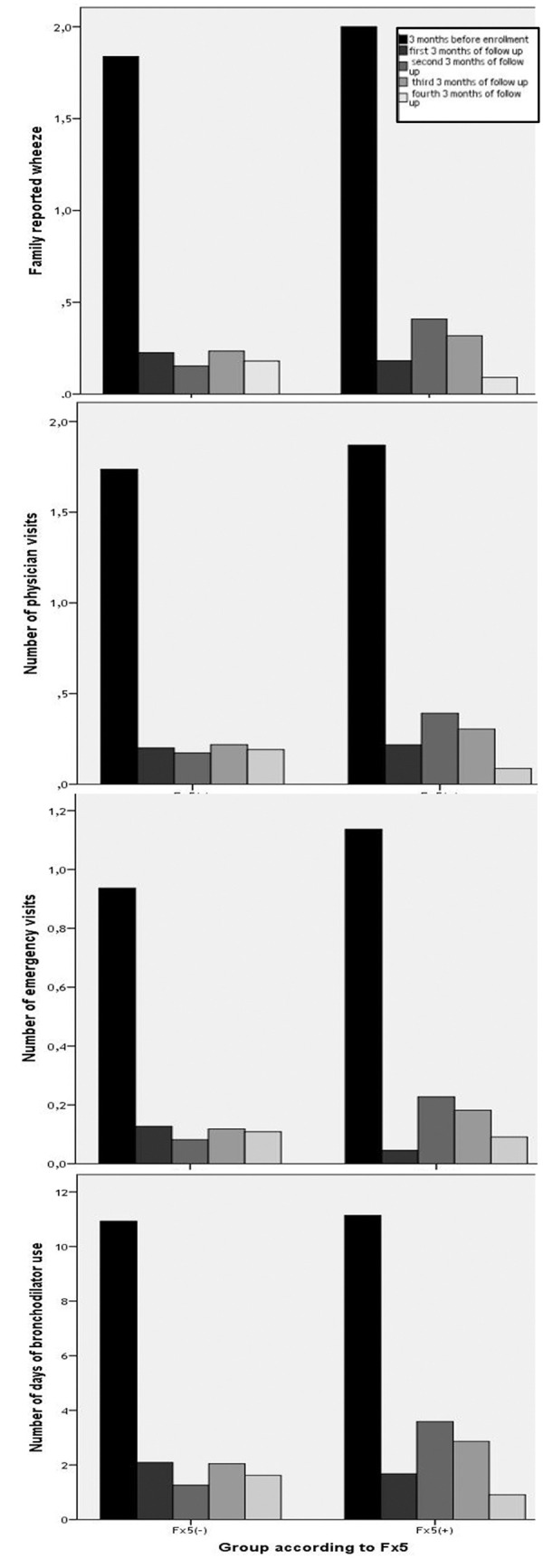
Wheezing prognosis indicators such as frequency of wheezing, number of emergency and physician visits and number of days of bronchodilator use in Fx5(+) and Fx5(–) subjects recorded every 3 months for the 1-year follow-up period.

Comparison of the groups at the end of every 3-month period during the 1-year follow up revealed that wheezing was significantly worse in the Fx5(+) group during the second 3-month period of follow-up. Number of wheezing episodes during this period was 0.4 ± 0.6 in the Fx5(+) group, while it was 0.1 ± 0.4 (p = 0.004) in the Fx5(–) group. Similarly, days of bronchodilator use were 3.3 ± 4.9 and 1.2 ± 3.3 in the Fx5(+) and Fx5(–) groups, respectively (p = 0.004) (Table 2) (Figure 2). 

Multivariable Poisson regression with food allergen sensitivity, age, and wheezing score at presentation revealed that Fx5 positivity is expected to change the yearly frequency of wheeze by a factor of 1.66 (p = 0.05, 95% CI 0.99 – 2.75). Similarly, age is expected to change the yearly frequency of wheeze by a factor of 0.95 (p = 0.005, 95% CI 0.92 – 0.99). Finally, a 1-point change in wheezing score at presentation changed the wheezing frequency in the following year by a factor of 1.11 (p = 0.005, 95% CI 0.67 – 1.99) (Table 3). 

### Levels of epithelial permeability and inflammation markers in the study population 

Levels of CC16 in the Fx5(+) group was 5.2 ± 2.4 ng/mL, while that in the Fx5(–) group was 5.4 ± 3.2 ng/mL (p = 0.679). Similarly, levels of SP-D were not significantly different between the groups (161.4 ± 88.7 and 161.7 ± 101.8 ng/mL, p = 0.988) (Table 4). 

## Discussion 

In this prospective cohort study of children with recurrent wheezing, sensitization to food allergens, described as specific IgE positivity, was associated with an increased incidence of yearly wheezing episodes irrespective of the presence of clinical food allergy. Moreover, younger age and wheezing severity were associated with increased frequency of wheezing during follow-up. On the other hand, serum CC16 as a marker of airway epithelial permeability and SP-D as a marker of inflammation were not significantly different between children with and without sensitization to food allergens. 

Specific IgE positivity to specific food antigens such as egg white and milk as well as inhalant antigens in wheezing children younger than 2 years of age has been found to be associated with asthma at later ages [10]. Similarly, we found that specific IgE positivity to common food allergens is associated with more frequent recurrence of wheezing in 1 year follow-up in children younger than 3 years. Moreover, sensitization to both aero and food allergen has been shown to be predictive of asthma development in the Cincinnati Childhood Allergy and Air Pollution Study, a cohort of more than 700 children. Skin prick test sensitivity to more than 1 allergen nearly doubled the risk of asthma development [11]. Allergy to more than 2 food antigens was found to be associated with lower FEF_25-75_ values in asthmatic children non sensitized to aeroallergens [12]. An adult study on asthmatics demonstrated that sensitization to food allergens was associated with higher exhaled nitric oxide (FeNO) and periostin levels suggesting higher type 2 inflammation [13]. Moreover, adolescents with asthma and clinically diagnosed food allergy were found to have higher FeNO [14]. In concordance with the results of the above-mentioned studies, our results demonstrated that sensitization to food allergens irrespective of clinical food allergy findings is associated with more severe wheezing in children with recurrent wheezing. 

CC16 is a 16-KDa protein secreted by non-ciliated bronchial epithelial cells [15]. CC16, is proposed to be a marker to detect permeability changes of the lung epithelial barrier and integrity of Club cells. It can be detected in bronchoalveolar lavage, epithelial lining fluid (ELF), and serum. Its concentrations in serum of nonsmokers are ~ 10 – 15 µg/L which is much lower than that in ELF leading to leakage from respiratory tract into serum [16]. It has been proposed that depletion of CC16 due to Club cell damage or due to intravascular leakage of the protein through damaged epithelium may play a critical role in development of lung inflammation and injury. There are three important mechanisms that may lead to increased concentrations of CC16 in serum: increased intravascular leakage through damaged lung epithelial barrier, decreased production from Club cells, and reduction in clearance from plasma with renal insufficiency [15]. CC16 mRNA expression from bronchial epithelial cells were found to be negatively correlated with Th2 genes such as IL1R but positively correlated with Th1 genes such al IL12 in subjects with asthma. Moreover, low expression of CC16 was associated with asthma severity [17]. These important features of CC16 might indicate a potential role as an epithelial barrier biomarker in wheezing children. On the other hand, we did not detect a significant difference of CC16 in wheezing children with and without sensitization to food allergens. Further research on local epithelial barrier markers in wheezing children may be warranted. 

SP-D is a lung collectin expressed in alveolar epithelial cells and epithelium of proximal airways. Expression of SP-D increases in response to inflammation and is a cellular modulator of immune response in the lung [5]. Serum levels of this protein was found to be higher in asthmatic subjects compared to the controls and proposed to be associated with small airways disease [18]. House dust mite and pet sensitization was found to be associated with higher SP-D levels in adolescents [19]. Moreover, higher SP-D levels were detected in subjects with persistent asthma and more peripheral airway dysfunction [20]. Similarly, respiratory syncytial virus bronchiolitis cases with more severe clinical findings were shown to have higher SP-D levels suggesting SP-D to be a valuable marker or lung injury [21]. However, our results failed to demonstrate a significant difference of SP-D levels in wheezing children with and without sensitization to food allergens. This may be attributed to the variation of SP-D levels according to airway inflammatory status. 

A major limitation of this study was loss to follow-up; of the 295 enrolled subjects, 133 (45%) presented for 12-month follow-up. We tested for potential biases due to loss to follow-up by comparing the baseline characteristics such as age and Fx5 positivity in children followed up for 12 months versus the ones lost to follow-up and we did not detect a significant difference. Another limitation was the partially objective nature of family-reported wheezing episodes that depends on parental perception. We tried to overcome this by asking them to present to the outpatient department when they felt wheezing and we added objective prognostic variables included number of wheezing episodes, number of emergency visits for wheezing, number of days of systemic steroid use and number of days of bronchodilator use. The strength of this study is the cohort design that allowed us to evaluate a possible causal relationship between sensitization to food allergens irrespective of food-associated clinical allergy findings and new-onset wheezing episodes. 

In conclusion, recurrence and duration of wheezing is more severe in children with sensitization to food allergens defined as serum specific IgE positivity irrespective of food-associated clinical allergy findings. This finding is important for prediction of prognosis in children with recurrent wheezing. Further research is required to investigate the potential role of food elimination in these children who have frequent wheezing episodes with food allergen specific IgE positivity. We were not able to demonstrate our initial hypothesis that CC16 levels as a marker of impaired airway epithelial barrier permeability associated with sensitization to allergens may be different between children with and without sensitization to food allergens. Moreover, SP-D, which is an inflammatory marker, was not found to be significantly different in wheezing children with sensitization to food allergens. 

## Authors’ contributions 

Ozge Yilmaz: Investigation, resources, writing original draft, methodology, formal analysis, project administration. Cecilia M. Patino: Methodology, formal analysis, writing-review and editing. Hasan Yuksel: Conceptualization, writing-review and editing, supervision. Fatma Taneli: Investigation, methodology, writing-review and editing. Esra Toprak Kanik: Patient data collection Ahmet Turkeli: Patient data collection. Ceyhun Gozukara: Biochemical analysis. Sezen Irmak. Biochemical analysis. 

## Funding 

This research has been funded by the American Thoracic Society MECOR award. 

## Conflict of interest 

The authors declare that they have no conflict of interest related to the manuscript. 

**Table 1.** Baseline characteristics of the study population across food allergy sensitivity status (n = 295).

	**Fx5(+) ** **n = 44**	**Fx5(–) ** **n = 251**	**p-value**
Age (months)^†^	20.4 ± 9.5	17.8 ± 8.9	0.09*
Sex (males)^‡^	31 (70.5)	171 (68.1)	0.86**
Number of siblings^†^	0.9 ± 0.8	0.7 ± 0.8	0.05*
Wheezing severity score^§^	3.1 ± 2.8	2.9 ± 2.5	0.58*
Number of wheezing episodes^¶^	1.8 ± 0.9	1.8 ± 0.9	0.69*
Number of physician visit^¶^	1.8 ± 1.0	1.7 ± 0.8	0.43*
Number of emergency visits^¶^	1.1 ± 1.0	0.9 ± 0.8	0.46*
Days of bronchodilator use^¶^	11.2 ± 5.7	11.2 ± 5.9	0.99*
Total IgE levels^§^	250 (192)	66.7 (110)	< 0.001*

^†^Expressed as mean(standard deviation); ^‡^number of males expressed as n(%) in each group; ^§^at enrollment; expressed as mean ± standard deviation; ^¶^in the 3 months period before enrollment; expressed as mean ± standard deviation; *independent samples t-test; **Fisher’s exact test.

**Table 2.** Frequency of disease and clinical outcomes at 3, 6, 9, and 12 months across food allergen sensitivity status†.

	**3 months ** **n = 248**	**6 months ** **n = 195**	**9 months ** **n = 154**	**12 months ** **n = 133**
Number of wheezing episodes				
Fx5(+)	0.3 ± 0.6	0.4 ± 0.6	0.3 ± 0.5	0.1 ± 0.3
Fx5(–)	0.2 ± 0.5	0.1 ± 0.4	0.2 ± 0.4	0.2 ± 0.4
p*	0.421	0.004	0.72	0.31
Number of physician visits				
Fx5(+)	0.2 ± 0.4	0.4 ± 0.6	0.3 ± 0.5	0.1 ± 0.3
Fx5(–)	0.2 ± 0.5	0.2 ± 04	0.2 ± 0.4	0.2 ± 0.4
p*	0.892	0.013	0.606	0.257
Number of emergency visits				
Fx5(+)	0.1 ± 0.3	0.2 ± 0.5	0.2 ± 0.4	0.1 ± 0.3
Fx5(–)	0.1 ± 0.4	0.1 ± 0.3	0.1 ± 0.3	0.1 ± 0.3
p*	0.828	0.007	0.617	0.816
Days of bronchodilator use				
Fx5(+)	1.9 ± 3.8	3.3 ± 4.9	2.4 ± 4.1	0.9 ± 1.9
Fx5(–)	2.1 ± 4.7	1.2 ± 3.3	2.1 ± 3.9	1.6 ± 3.9
p*	0.853	0.004	0.724	0.418

^†^Values expressed as mean ± standard deviation; *independent samples t-test.

**Table 3.** The association between food sensitivity and yearly frequency of wheezing in children adjusted for age and wheezing score at baseline*.

	**IRR**	**95%CI†**	**p**
Fx5 positivity (+ vs. –)	1.66	0.99 – 2.75	0.05
Age in months	0.95	0.92 – 0.99	0.005
Wheezing score at baseline	1.11	1.03 – 1.19	0.008

*Poisson regression analysis; ^†^95% confidence interval of the difference; IRR = incidence rate ratios.

**Table 4.** Club cell protein-16 and surfactant protein-D levels of the study subjects at enrollment.

	**Fx5(+) ** **n = 161**	**Fx5(–) ** **n = 33**	p (95%CI)*
Club cell protein-16 (ng/mL)^†^	5.2 ± 2.4	5.4 ± 3.2	0.679 (–0.9 – 1.4)
Surfactant protein-D (ng/mL)^†^	161.4 ± 88.7	161.7 ± 101.8	0.988 (–37.3 – 37.9)

^†^Values expressed as mean – standard deviation; *independent samples test (95% confidence interval of the difference).
